# Supplementary material for: Double-Stranded RNA Structural Elements Holding the Key to Translational Regulation in Cancer: The Case of Editing in RNA-Binding Motif Protein 8A
Source: Cells. 2021 Dec 15;10(12):3543. doi: 10.3390/cells10123543 (PMC8699885; doi:10.3390/cells10123543)
Supplement: Supplementary file 1 [file cells-10-03543-s001.zip › cells-1489391 Supplementary material/Figures S1-S8.pdf]

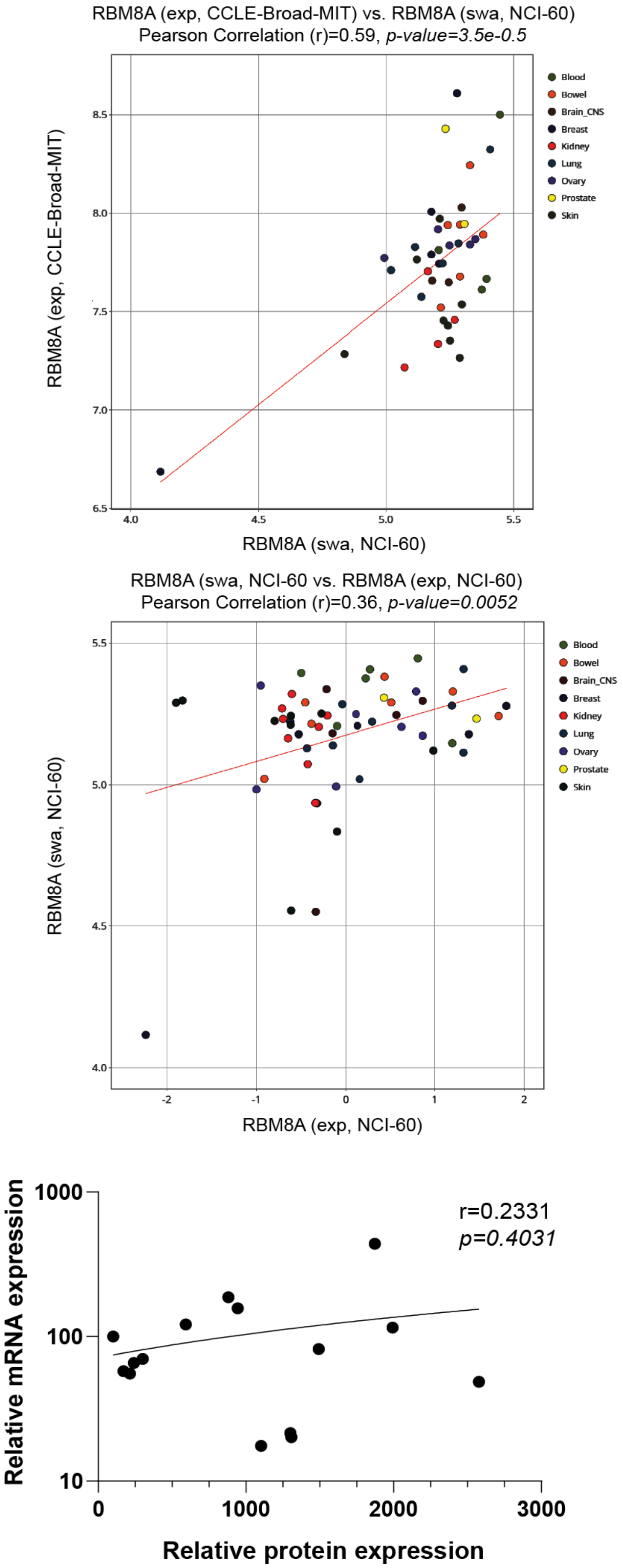
A
RBM8A (exp, CCLE-Broad-MIT) vs. RBM8A (swa, NCI-60)
Pearson Correlation (r)=0.59, p-value=3.5e-0.5
RBM8A (exp, CCLE-Broad-MIT)
RBM8A (swa, NCI-60)
Blood
Bowel
Brain_CNS
Breast
Kidney
Lung
Ovary
Prostate
Skin
RBM8A (swa, NCI-60 vs. RBM8A (exp, NCI-60)
Pearson Correlation (r)=0.36, p-value=0.0052
RBM8A (swa, NCI-60)
RBM8A (exp, NCI-60)
B
r=0.2331
p=0.4031
Relative mRNA expression
Relative protein expression

Figure S1. Correlation of RBM8A protein and RNA expression in different human cell lines. (**A**). Data were obtained using CellMinerCDB (https://discover.nci.nih.gov/rsconnect/cellminercdb/, accessed on 28 April 2020). Upper graph: RBM8A protein expression (NCI-60 dataset) on x-axis and mRNA expression (CCLE-Broad-MIT dataset) on y-axis. Lower graph: RNA expression on x-Axis (NCI-60 dataset) and protein expression on y-axis (NCI-60 dataset). (**B)** Correlation of RBM8A protein and RNA expression in mesothelioma cell lines.

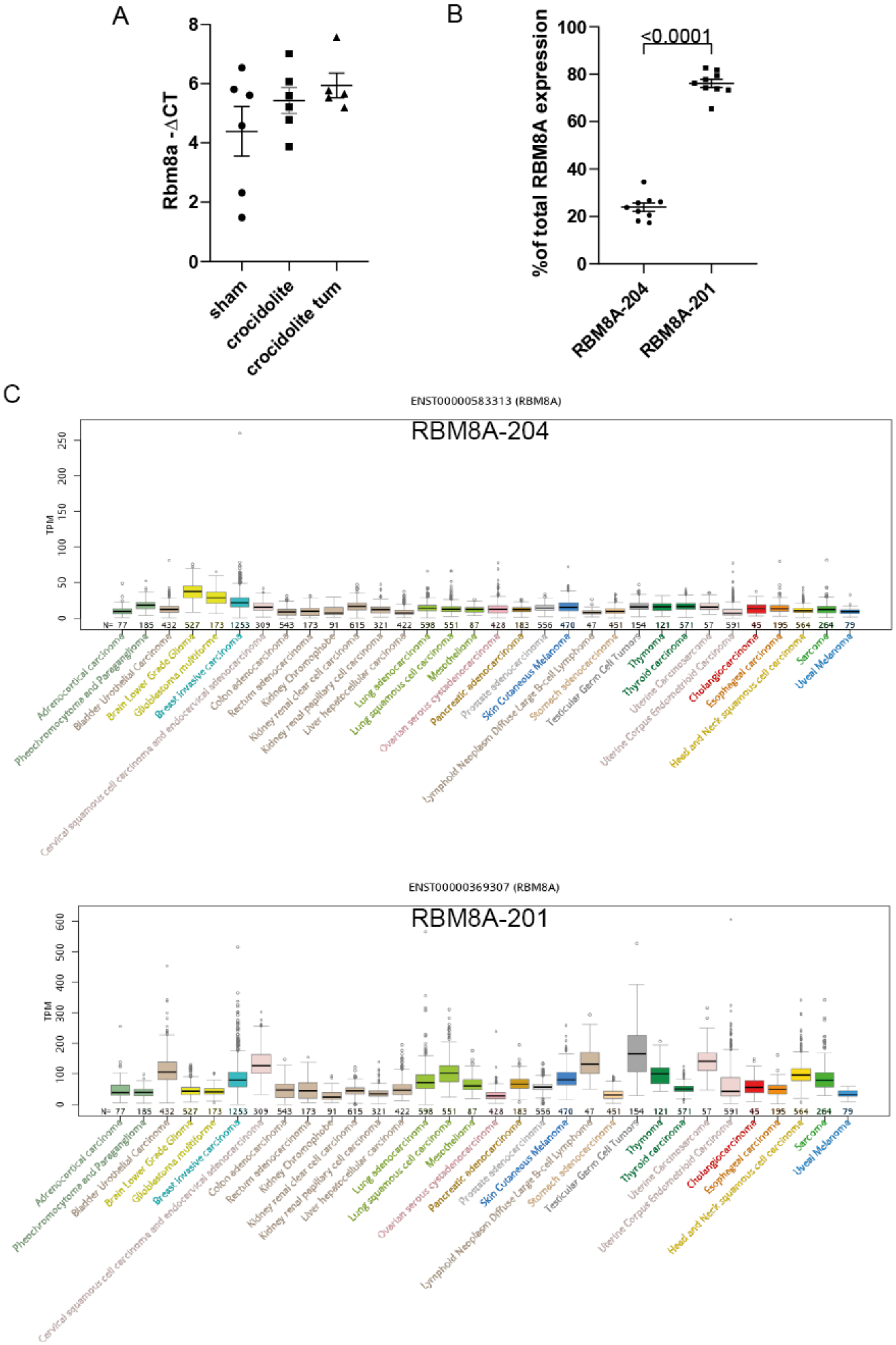

Figure S2. *Rbm8a* mRNA expression in crocidolite (blue asbestos) exposed mice and abundance of *RBM8A* transcripts in mesothelial cells or various cancers. (**A**). *Rbm8a* expression levels do not vary significantly upon crocidolite exposure. (**B**)

RBM8A-204 levels are significantly lower compared to *RBM8A-201* levels in in mesothelial cells. C. Expression profile of *RBM8A-204* (upper panel) and RBM8A -201 (lower panel) in different cancer types. TPM=Transcripts per Million.

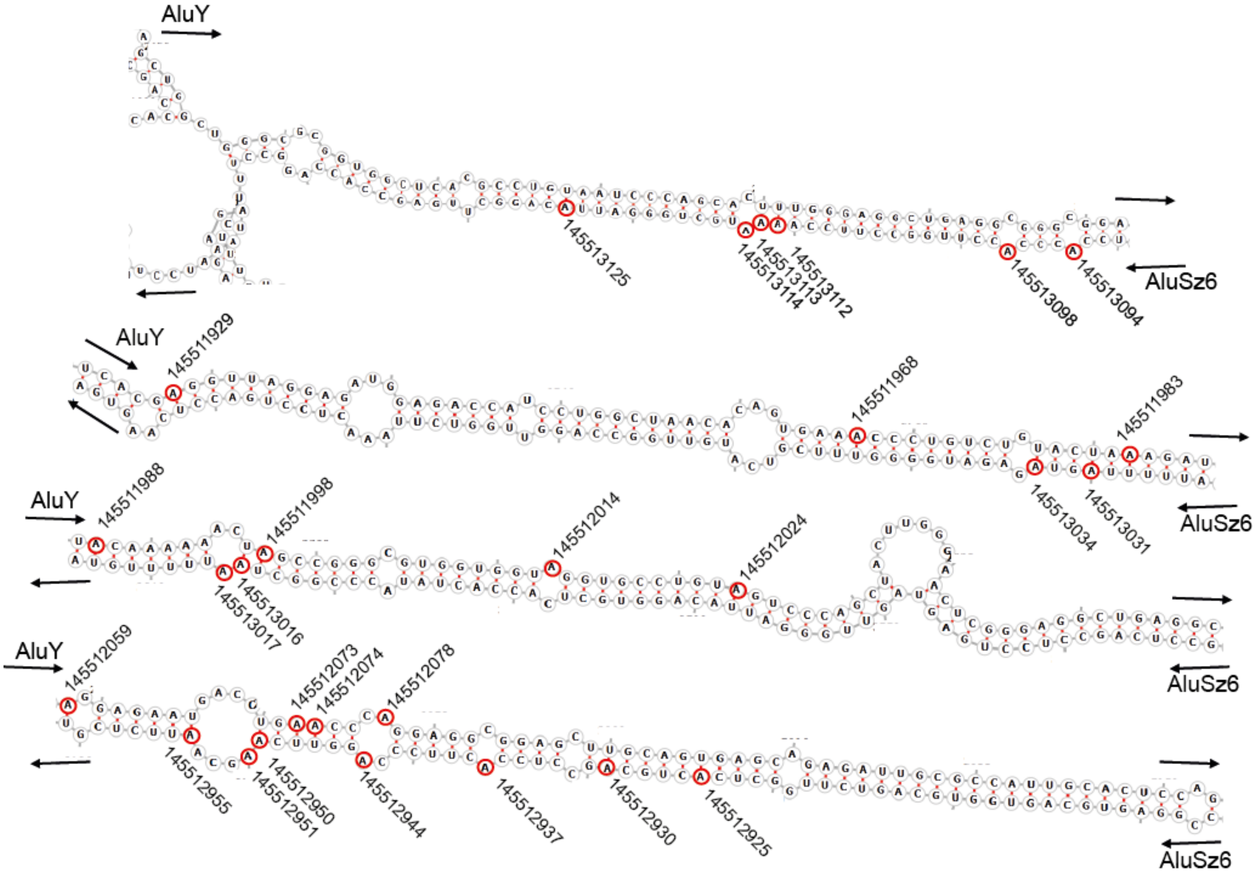

Figure S3. dsRNA stretch of RBM8A 3'UTR formed by AluY and AluSz6 elements. Red circles indicate A-I editing sites and the number refers to nucleotide positions on chromosome 1, hg19. Structure is predicted by RNAfold webserver (http://rna.tbi.univie.ac.at//cgi-bin/RNAWebSuite/RNAfold.cgi, accessed on 27 April 2021).

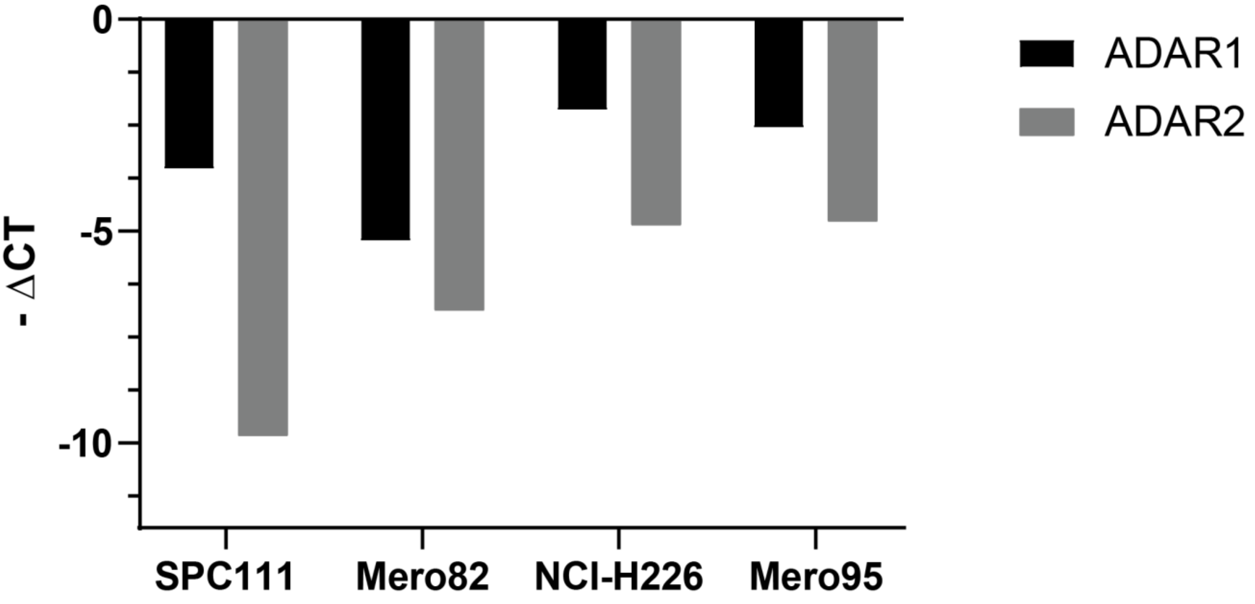

Figure S4. ADAR1/2 mRNA heterogeneous expression in four MPM cell lines. *ADAR1* (black) and *ADAR2* (gray) mRNA levels in SPC111, Mero82, NCI-H226 and Mero95 MPM cell lines.

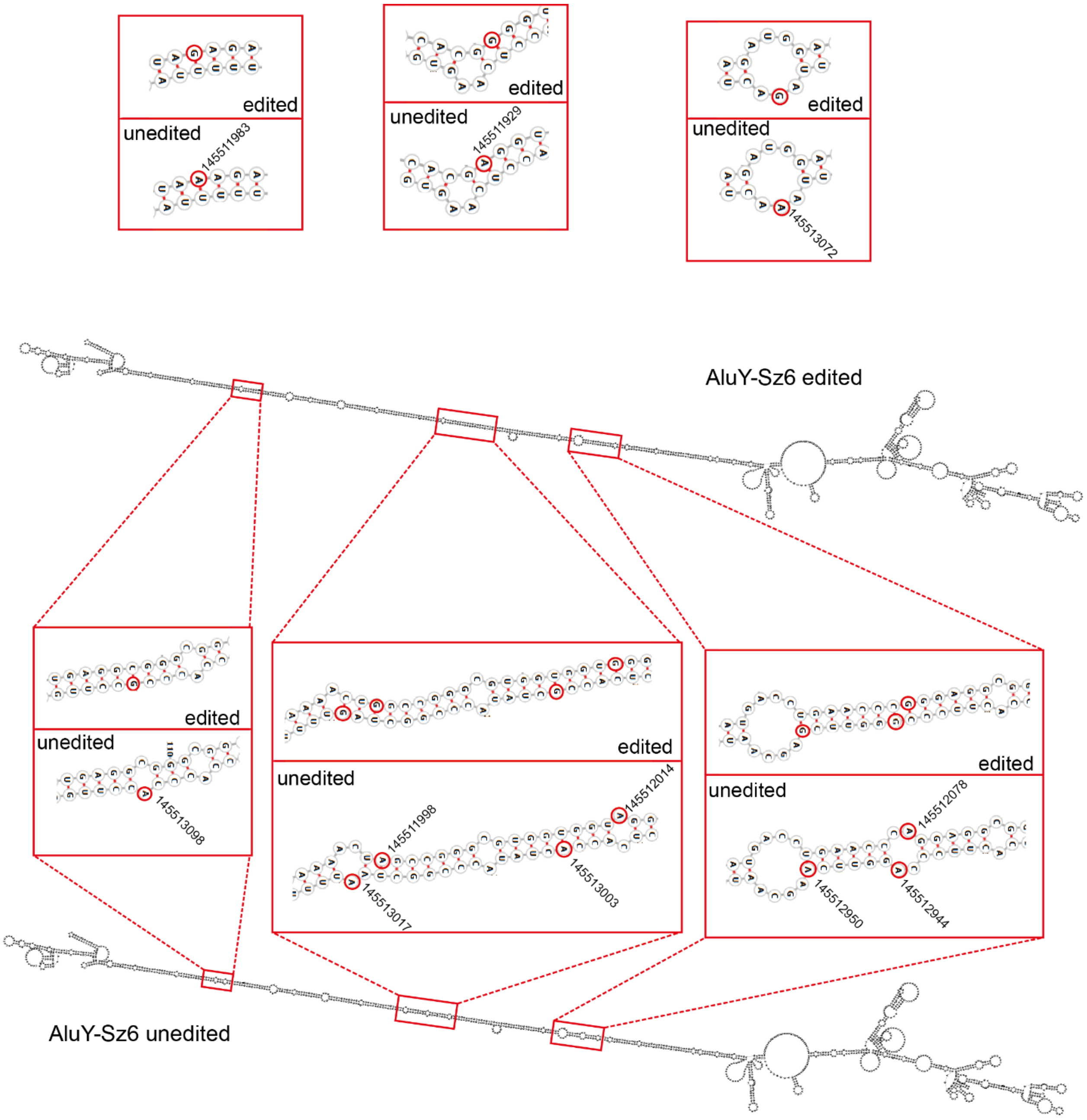

Figure S5. Editing of *RBM8A* 3′UTR AluY-AluSz6 entails structural changes. Red squares in lower part magnify sections with structure change upon editing. Red circles indicate editing sites and numbers of the nucleotide position in chromosome 1, hg19. In the upper part are shown some edited sites leading to no structural change. Structures are predicted by RNAfold webserver (http://rna.tbi.univie.ac.at//cgi-bin/RNAWebSuite/RNAfold.cgi, accessed on 27 April 2021).

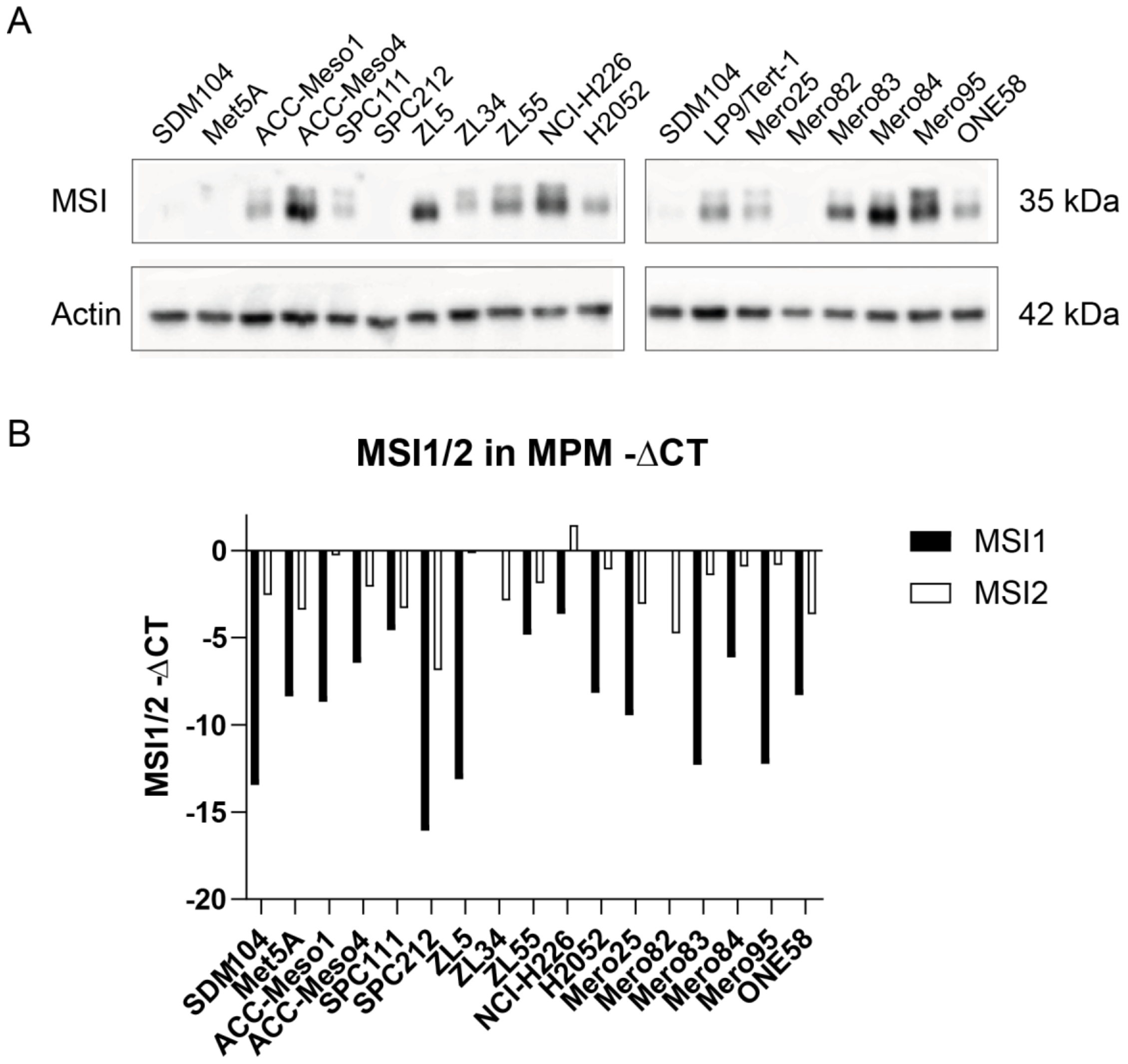

Figure S6. MSI2 expression is higher compared to MSI1 in MPM. (**A**). Western Blot analysis of MSI protein expression in 16 MPM cell lines and mesothelial cells. Actin is used as loading control. (**B)** Relative mRNA levels for *MSI1* (black) and *MSI2* (white).

```
Gibbon          ggctgggcgcggtggctcaagcctgtaatcccagcactttgggagggtgaggcgggcgga 60
Orangutan       ggctgggcgcggtggctcacgcctgtaatcccagcactttgggaggctgaggtgggtgga 60
Bonobo          ggctgggcgcggtggctcacgcctgtaatcccagcactttgggaggctgaggcgggcgga 60
Human           ggctgggcgcggtggctcacgcctgtaatcccagcactttgggaggctgaggcgggcgga 60
                ****************** *************************** ***** *** ***

Gibbon          tcacgaggtcaggagatcgagaccatcccggctaacacagtgaaaccccgtctgtactaa 120
Orangutan       tcacgaggtcaggagatggagaccatcctggctaacacagtgaaaccccatctgtactaa 120
Bonobo          tcacgaggttaggagatggagaccatcctggctaacacagtgaaaccctgtctgtactaa 120
Human           tcacgaggttaggagatggagaccatcctggctaacacagtgaaaccctgtctgtactaa 120
                ********* ******* ********** *******************  **********

Gibbon          agatagaaaaaattagctgggcgtggtggcgggcgcctgtagtcccagctactggggaac 180
Orangutan       agatacaaaaaactagccgggcgtagtggcgggcacctgtagtcccagctactcgggaac 180
Bonobo          agatacaaaaaactagccgggcgtggtggtaggtgcctgtagtcccagctacttgggaac 180
Human           agatacaaaaaactagccgggcgtggtggtaggtgcctgtagtcccagctacttgggaac 180
                ***** ****** **** ****** ****  **  ****************** ******

Gibbon          tcgggaggctgaggcaggagaatggcctgaacccaggaggcggagcttgcagtgagccga 240
Orangutan       ttgggaggctgaggcaggcgaatggcctgaacccaggaggtggagcttgcagtgagcaga 240
Bonobo          tcaggaggctgaggcaggagaatgacctgaacccaggaggcggagcttgcagtgagcaga 240
Human           tcgggaggctgaggcaggagaatgacctgaacccaggaggcggagcttgcagtgagcaga 240
                *  *************** ***** *************** **************** **

Gibbon          gattgcgccactgcactccagcctgggcgtcagagtgagactcagtctccaaaaaaaaaa 300
Orangutan       gattgcgccattgcactccagcctgggcgacagagtgagactgtctccaaaaaaa----a 296
Bonobo          gattgcgccattgcactccagcctgggcgacagagtaagactgtgtctccaaaaa----a 296
Human           gattgcgccattgcactccagcctgggcgacagagtaagactgtctccaaaaaaa----a 296
                ********** ****************** ****** *****   **   *****    *

Gibbon          aaaaaaaaatagtcaaagctcttggatttacagtttggtccacagccttgttttgatctt 360
Orangutan       ------aaaaagtcaaagatcttggatttatagtttggtccacagccttgttttgatctt 350
Bonobo          aaaaataaataatcaaagctcttggatttatagtttggtccacagccttgttttgatctt 356
Human           aaaaaataataatcaaagctcttggatttatagtttggtccacagccttgttttgatctt 356
                       ** * ****** *********** *****************************

Gibbon          tcctttctcctgttttattgccatttaccacgtactgtagaaacatccctttcaactgct 420
Orangutan       tcctttctcctgttttattgccatttaccacgtactgtagaaacatccctttcaactgct 410
Bonobo          tcctttatcctgttttattgccatttaccatgtactgtagaaacatccctttcaactgct 416
Human           tcctttatcctgttttattgccatttaccacgtactgtagaaacatccctttcaactgct 416
                ****** *********************** *****************************
```

```
Gibbon          gataacttggaaacaagcctacaaaaataagtaatttctaactactactaatgctacccc 480
Orangutan       gataacttggaaacaagcctacaaaaataagtaatttctaactattcctaatactacccc 470
Bonobo          gataacttggaaacaagcctacaaaaataagtaatttctaactactcctaatactaccta 476
Human           gataacttggaaacaagcctacaaaaataagtaatttctaactactcctaatactaccta 476
                ******************************************** * ***** *****

Gibbon          taactacccctaagcccttaccacactaacgtgacattattaaatttttattttattaa 540
Orangutan       taactacccctaagcccttaccacactaatgtgacattattaaatttttattttattaa 530
Bonobo          taactacccctaagcccttaccactctaacgtgacattattaaatttttattttattaa 536
Human           taactacccctaagcccttaccactctaacgtgacattattaaatttttattttattaa 536
                ************************ **** ******************************

Gibbon          cactaatatttaactacaattacagcatatgggcaatgcagaatttacccaaaaggata 600
Orangutan       cactaatatttaactacaattacagcatatgggcaatgcagaatttacctaaaaggata 590
Bonobo          cactaatatttaactacaattacagcatatgggcaatacagaatttacctaaaaggata 596
Human           cactaatatttaactacaattacagcatatgggcaatacagaatttacctaaaaggata 596
                ************************************** *********** *********

Gibbon          ctaatttggaactaaaaataacacctttttgcacatgtatcatgtcacaaccagtttgcc 660
Orangutan       ctaatttggaaccaaaaaatatcacctttttgcacatgtatcatgtcacaaccagtttgcc 650
Bonobo          ctaatttggaacaaaaaaaaatcacctttttgcacatgtatcatgtcacaaccagtttgcc 656
Human           ctaatttggaacaaa-aaaaatcacctttcgcacatgtatcatgtcacaaccagtttgcc 655
                ************ ** *** * ******* ******************************

Gibbon          attgaaacaaatagaggttgcaaatattgtcagattgtcaggctgtaagaaaggatgaaa 720
Orangutan       attgaaacaaatagaggttgcaaatattgtcagattgtcaggctgtaagaaaggatgaaa 710
Bonobo          attgaaacaaatagaggttgcaaatattgtcagattgtcaggctgtaagaaaggatgaaa 716
Human           attgaaacaaatagaggttgcaaatattgtcagattgtcaggctgtaagaaaggatgaaa 715
                ************************************************************

Gibbon          ttcatttcccattgcatcatcttgtggcccatggatttcaagtgccttagccaaaatcgt 780
Orangutan       ttcatttcccattgcatcatcttgtggcccatggatttcaagtaccttagccaaaatcat 770
Bonobo          ttcatttcccattgcatcatcttgtggcccatggatttcaagtgccttagccaaaatcat 776
Human           ttcatttcccattgcatcatcttgtggcccatggatttcaagtgccttagccaaaatcat 775
                ******************************************* ************** *

Gibbon          atagctagttagcagtagagctgagactcagaaaaa---aaagtaaaacagacagactga 837
Orangutan       atagctagttagcagtagagccgagactcagaaaaaaccaaagtaaaacaggcagactga 830
Bonobo          atagctagttagcagtagagccgagactcagaaaaaaaacaaagtaaaacaggcagactga 836
Human           atagctagttagcagtagagccgagactcagaaaaaaaacaaagtaaaacaggcagactga 835
                ********************* **************   ************ ********
```

```
Gibbon         aacaaaaagtcttctaattcccagtccccgtgtaaaatttgcttcatataaacaaacctg 897
Orangutan      aacaaaaagtcttctaattcccagtccacatgtaaaatttgcttcatataaacaaaccta 890
Bonobo         aacaaaaagtcttctaattcccagtccacatgtaaaatttgcttcatataaacaaaccta 896
Human          aacaaaaagtcttctaattcccagtccacatgtaaaatttgcttcatataaacaaaccta 895
               *************************** * *****************************

Gibbon         attgtaaatggcactgtagcaacaggcttctttttaacagttggattggtaaaggtcttg 957
Orangutan      attgtaaatggcactgtagcaacaggcttctttttaacacttggattggtaaaggccttg 950
Bonobo         attgtaaatggcactgtagcaacaggcttctttttaacacttggattggtaaaggtcttg 956
Human          attgtaaatggcactgtagcaacaggcttctttttaacacttggattggtaaaggtcttg 955
               *************************************** *************** ****

Gibbon         tttgcaacatattgcaagtattatttttctctcttttttttttt--gttttccccaacag 1015
Orangutan      tttgcaacatattagaagtattatttttctctcttccccccctacccccacccccaacag 1010
Bonobo         tttgcaacatattagaagtattatttttctcttttccccc-----ccccacccccaacag 1011
Human          tttgcaacatattagaagtattatttttctctttccccccc---accccacccccaacag 1012
               *************  ***************** *                ********** 

Gibbon         agtctggctctgtcgcccacgctggagtgcagtggtgcagtcttggctcactgcagcctc 1075
Orangutan      agtctggctctgtcgcccaccccggagtgcagtggtgcagtcttggctcactgcagcctc 1070
Bonobo         agtctggctctgccgcccacgccggagtgcagtggtgcaatcttggctcactgcagcctc 1071
Human          agtctggctctgccgcccacgccggagtgcagtggtgcagtcttggctcactgcagcctc 1072
               ************ ******* * **************** ********************

Gibbon         cacctcccaggttcaagcaattctcgtgcctcagcctcctgagtagttgggattacaggt 1135
Orangutan      cacttcccaggttcaagcaattctcgtgcctcagcctcctgagtagttgggattacaggt 1130
Bonobo         cacttcccaggttcaagcaattctcgtgcctcagcttcctgagtagttgggattacaggt 1131
Human          cacttcccaggttcaagcaattctcgtgcctcagcctcctgagtagttgggattacaggt 1132
               *** ******************************* ************************

Gibbon         gctcactactacacccggctaatttttgtatttttagtagagatggggtttcgtcatgtt 1195
Orangutan      gctcaccactacacccggctaatttttgtatttttagtagagatggggtttcgtcatgtt 1190
Bonobo         gctcaccactatacccggctaatttttgtatttttagtagagatggggtttcgtcatgtt 1191
Human          gctcaccactatacccggctaatttttgtatttttagtagagatggggtttcgtcatgtt 1192
               ****** **** ************************************************

Gibbon         ggccaggttggtcttgaactcctgacctcaagtgattcacccaccttggccttccaaaat 1255
Orangutan      ggccaggttggtcttgaactcctgacctcaagtgatccacccaccttggccttccaaaat 1250
Bonobo         ggccaggttggtcttaaactcctgacctcaagtgatccacccaccttggccttccaaaat 1251
Human          ggccaggttggtcttaaactcctgacctcaagtgatccacccaccttggccttccaaaat 1252
               *************** ******************** ***********************
```

```
Gibbon        gctgggattacaggcttgagccaccaggcc    1285
Orangutan     gctgggattacaggcttgagccaccaggcc    1280
Bonobo        gctgggattacaggcttgagccaccaggcc    1281
Human         gctgggattacaggcttgagccaccaggcc    1282
              ******************************
```

AluY

AluSz

Figure S7. AluY-AluSz6 sequence is highly conserved between humans and primates**.** *RBM8A* 3'UTR sequence containing AluY and AluSz6 elements compared between human, bonobo, orangutan and gibbon using Clustal Omega alignment tool (https://www.ebi.ac.uk/Tools/msa/clustalo/, accessed on 23 September 2021). AluY is highlighted in yellow, AluSz6 in green. Stars indicate identical nucleotide in all species.

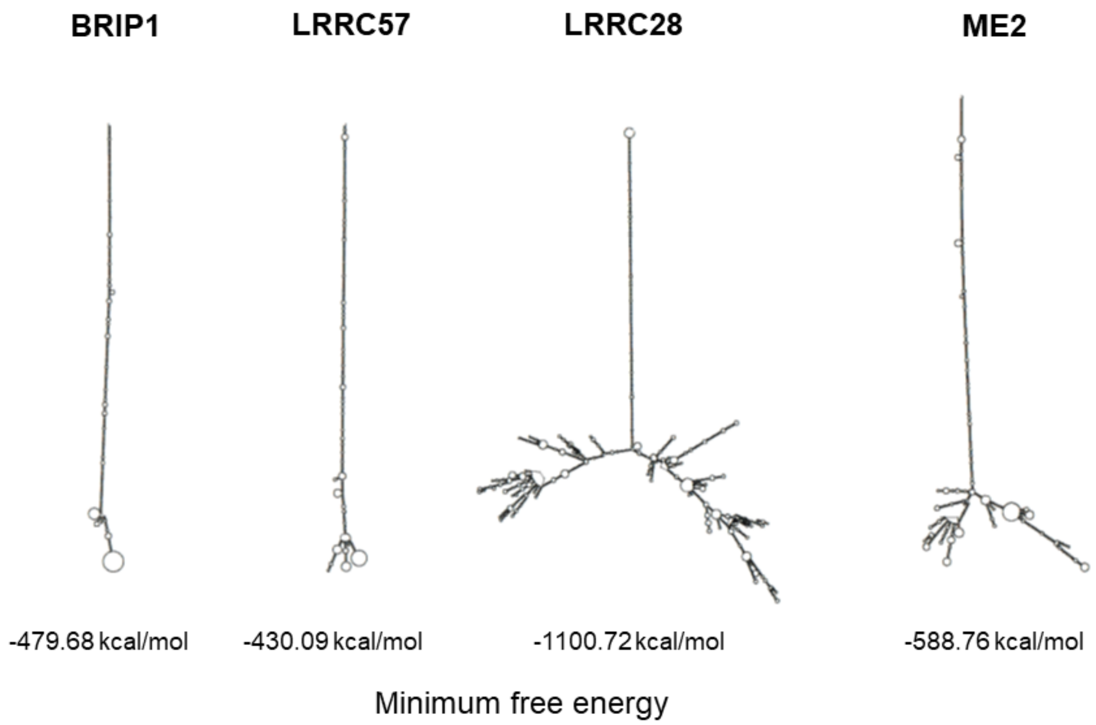

Figure S8. AluY-AluSz6 dsRNA structure is conserved in other transcripts enriched in the MPM translatome. Performing BLASTN using *RBM8A* 3'UTR sequence containing AluY and AluSz6 elements retrieved transcripts known to be edited by ADARs, to interact with MSI2 and to be present in the MPM translatome. They bear a similar dsRNA structure as RBM8A 3'UTR. BRIP1: BRCA1 interacting helicase 1, LRRC57: leucine rich repeat containing 57, LRRC28: leucine rich repeat containing 28, ME2: malic enzyme 2. Structures are predicted by RNAfold webserver (http://rna.tbi.univie.ac.at//cgi-bin/RNAWebSuite/RNAfold.cgi, accessed on 1 November 2021).
